# Recent development of ATP-competitive small molecule phosphatidylinostitol-3-kinase inhibitors as anticancer agents

**DOI:** 10.18632/oncotarget.12742

**Published:** 2016-10-18

**Authors:** Yu Liu, Wen-zhu Wan, Yan Li, Guan-lian Zhou, Xin-guang Liu

**Affiliations:** ^1^ School of Chemistry and Pharmaceutical Engineering, Qilu University of Technology, Jinan, P. R. China; ^2^ Department of Hematology, Qilu Hospital, Shandong University, Jinan, P. R. China

**Keywords:** phosphatidylinostitol-3-kinase, PI3K, inhibitor, mTOR pathway, anticancer

## Abstract

Phosphatidylinostitol-3-kinase (PI3K) is the potential anticancer target in the PI3K/Akt/ mTOR pathway. Here we reviewed the ATP-competitive small molecule PI3K inhibitors in the past few years, including the pan Class I PI3K inhibitors, the isoform-specific PI3K inhibitors and/or the PI3K/mTOR dual inhibitors.

## INTRODUCTION

**Figure 1:**
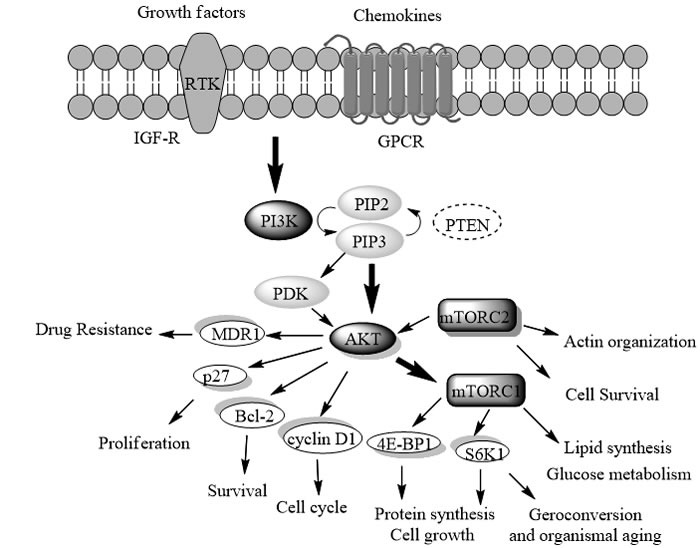
The PI3K/Akt/mTOR signaling pathway. Stimulation of this pathway is commonly triggered by the growth factors (e.g. IGF) or chemokine. Subsequent activation of the lipid PI3K leads to the phosphorylation of PIP2 to PIP3, which activates AKT and PDK1. Besides direct activation by PIP3, Akt could also be activated by PDK1 and mTORC2 (Rictor-mTOR). Then mTORC1 (Raptor-mTOR) was finally activated, which regulates cell growth, glucose and lipid metabolism, autophagy as well as protein synthesis, while mTORC2 regulates cell survival and actin reorganization. Additionally, the pathway is negatively regulated by PTEN.

The PI3K/AKT/mTOR signaling pathway (Figure 1) regulates diverse biological processes such as cell growth, cell proliferation, cell survival, protein synthesis, and glycolysis metabolism, which is frequently deregulated in human cancers [1–9]. PI3K (phosphatidylinostitol-3- kinase) is the main anticancer target within this pathway, and its correlation with tumor-genesis, progression and maintenance has been validated by several extensive studies [10–20].

To date, a total of eight PI3Ks have been identified, which are divided into four classes (I, II, III and IV) based on their sequence homology. As the most relevant to the PI3K/Akt/mTOR pathway (Figure 1), Class I PI3Ks are always referred to as PI3Ks [21]. Generally, Class I PI3Ks are further divided into IA and IB based on their different regulatory subunits and upstream activators [22]. Class IA PI3Ks are activated by RTKs and GPCRs, which contains three isoforms (PI3Kα, PI3Kβ and PI3Kδ) with the respective p110 catalytic subunit (p110α, p110β, and p110δ) bound to the p85 regulatory subunit [21]. Class IB PI3K consists of PI3Kγ, with the p110γ bound to p101 or p84, which is mainly activated by GPCRs such as chemokine receptors [23]. PI3Kα is known to play an important role in tumor genesis, which has been detected with persistent mutations and amplification in most human cancers including breast, ovarian, colorectal, stomach and gastric cancers [17, 24, 25]. PI3Kβ involves in the development of thrombotic diseases by activating platelets, while PI3Kγ and δ are the therapeutic targets of inflammatory and auto-immune diseases [22]. Beside PI3Kα, the other three isoforms (β, γ and δ) are also involved in tumor genesis, especially in the case of PTEN loss or inactivation. Moreover, as PI3K mutation and PTEN inactivation have been shown to be the causes of resistance to other targeted cancer therapies [26], the PI3K may even circumvent drug resistance to current chemotherapy in combination with other anticancer drugs [27].

## THE DEVELOPMENT OF PI3K INHIBITORS

The major PI3K inhibitors currently available are reversibly ATP-competitive. The X-ray crystal structure of PI3K [21] and those of its complexes with ATP, Wortmannin (1), LY294002 (3) [28, 29] and other diverse inhibitors facilitated and accelerated the development of PI3K inhibitors.

**Figure 2:**
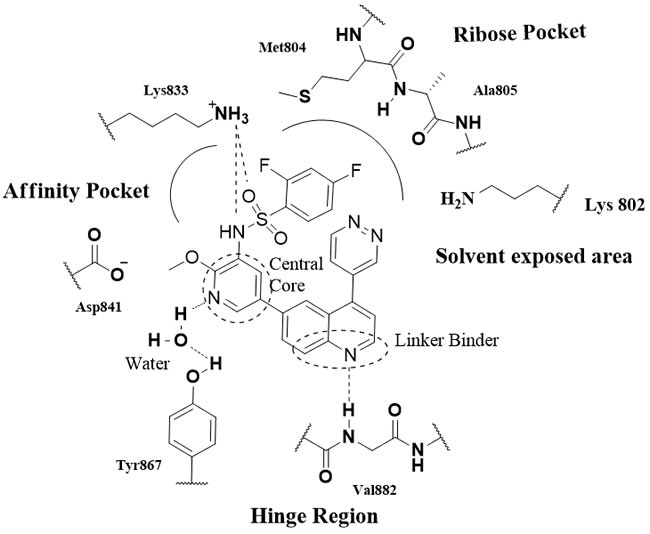
The potential interactions of P13K γ with GSK2126458

The binding models of inhibitors with the PI3K active site have also been generated in many recent studies. Overall, besides the solvent exposed area, the PI3K active site contains three key regions (PI3Kγ, Figure 2) [30]: the hinge region (Val882), the affinity pocket (Lys833, Asp841, Tyr867, Ala885, Ser806, Tyr867) or the back pocket (DFG-motif, gate keeper and catalytic lysine) and the ribose pocket (Met804, Ala805, Lys802, Met953, Asp964, Trp812, etc.). Accordingly, the ATP-competitive PI3K inhibitors mainly include (1) The hinge linker binder: substituents containing hydrogen donor/acceptor to interact with Val882 ( morpholine, pyperazine, indole, quinolone, amine, methoxy group, etc.) (2) The affinity pocket moiety: hydrophobic side chains or heterocycles, that may contain hydrogen donor/acceptor (sulfonamide, urea, pyridine, indazole, pyrazole, carbonyl group, etc.) forming H-bonds with the residues directly or aided by a water molecule (3) The ribose pocket moiety: various substituted lipophilic ring systems and groups ( pyrrolidine, pyrimidine, morpholine, thiomethyl, etc.) (4) The central core, cyclic or bicyclic, with diverse structure, having no obvious effects on the potency of the inhibitors ( e.g., pyrimidine, quinazoline, pyridine, quinolone, indole, pyrazines, quinoxalines, triazoles, imidazoles, thiazoles, etc.). Of them, the hinge region binder is crucial for PI3K inhibitors, while the affinity pocket interaction could lead to improved potency and potential selectivity.

Driven by efforts in computer-based rational drug design and SAR (Structure-Activity Relationship) studies, numerous promising PI3K inhibitors have been developed and a dozen of them have entered clinical trials for treatment of cancer or other diseases (Table 1) [19]. The first approved PI3K inhibitor Idelalisib (Gilead Sciences, Inc., also known as CAL-101 and GS-1101), an orally bioavailable PI3Kδ selective inhibitor with high potency and selectivity (p110δ IC_50_ = 2.5 nM), was approved by the FDA in July 2014 for the treatment of several hematological malignancies, in combination with rituximab.

**Table 1:** Major PI3K inhibitors that have entered into clinical trials

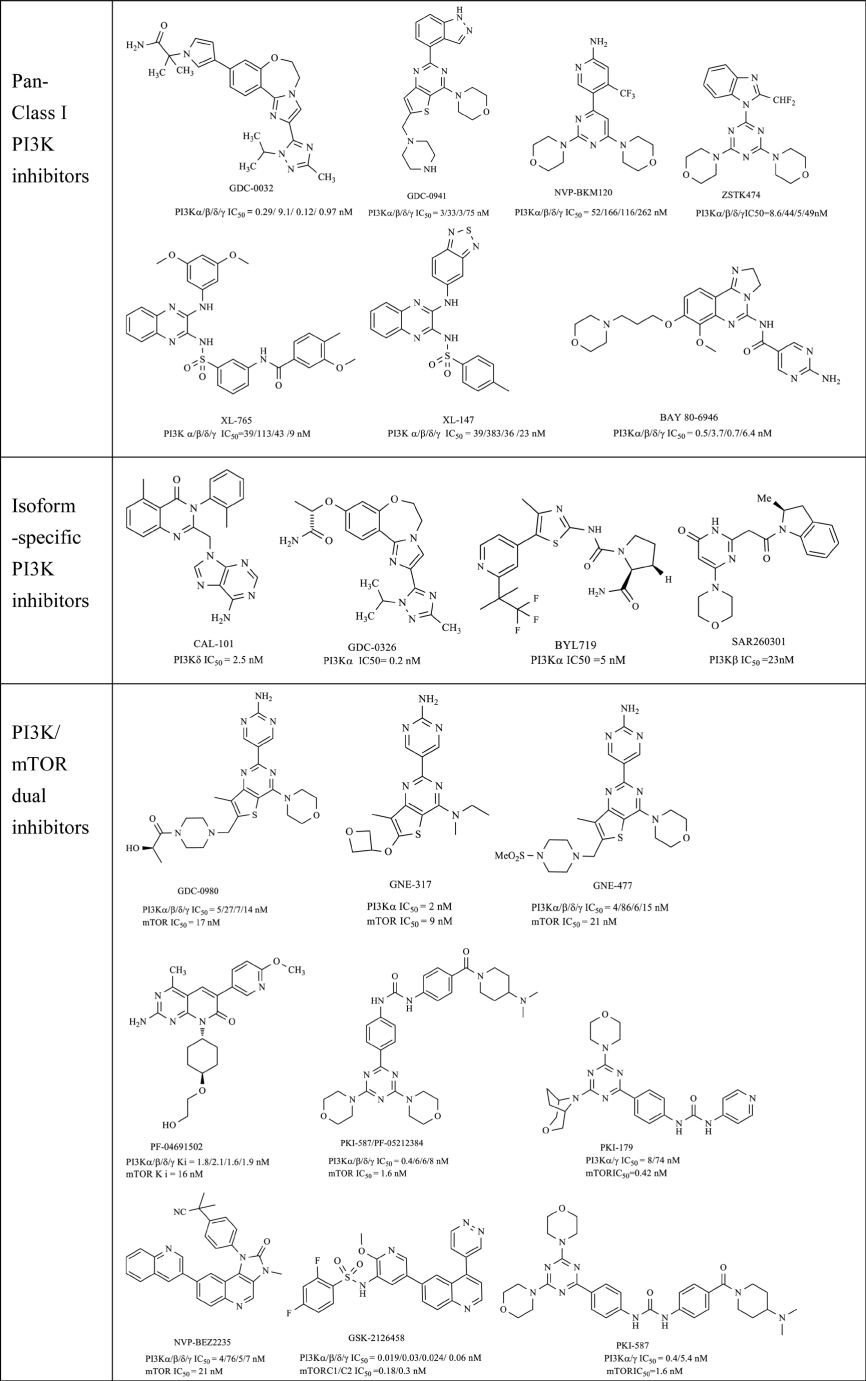

However, due to the high sequence homology of the catalytic domains and the conserved ATP-binding site, the key point for the development of PI3K inhibitors is to gain sufficient isoform- selectivity (δ and/or γ vs. α and β) and cross-kinase selectivity. Although it's not an easy task, the discovery of isoform-specific PI3K inhibitors were facilitated by the elucidation of the X-ray crystal structure of PI3K isoforms and those of its complexes with diverse inhibitors [27, 29, 30]. Additionally, because mTOR is PI3K-related kinase that has similar ATP site with PI3K, a number of PI3K inhibitors could also exhibit inhibitory activity against mTOR (PI3K/mTOR dual inhibitors), which may be more effective by delivering a powerful two-spot inhibition of the pathway and have the advantage of being less susceptible to PI3K drug resistance and abrogating the compensatory effects of mTOR inhibitors [31]. In fact, most of the drug candidates in this area were PI3K/mTOR dual inhibitors (Table 1).

In this review we provide a recent view about the PI3K inhibitors including the pan PI3K inhibitors, the isoform- specific PI3K inhibitors and the PI3K/mTOR dual inhibitors, as anticancer drugs in the PI3K/Akt/ mTOR pathway. As there are many well-written reviews in this field before, the novel PI3K inhibitors we emphasized here are those developed in the past eight years. Based on their core structures, the inhibitors were divided into five series: natural product derivatives; pyrimidines and quinazolines; pyridines, quinolines and indoles; pyrazines and quinoxalines; azoles and others.

## NATURAL PRODUCTS DERIVATIVES

Wortmannin (1), a steroidal furan derivative isolated from Penicillium wortmanni, was a pan PI3K inhibitor (IC_50_ at 50 μM ATP~4.0, 0.7, 4.1, 9.0 nM for PI3K α, β, δ and γ, respectively). At higher concentrations, it could also inhibit irrelevant kinases and PI3K-related kinases, such as mTOR, DNA-PK, and ATM.

The quercetin (2), a flavonoid, is a moderate pan PI3K inhibitor (PI3Kα IC_50_~3.8 μM). Diverse substitution of the chromone core of quercetin was studied and LY294002 (3), with a morpholine moiety replacement for the catechol ring, was identified by researchers at Lilly. Compound (3) was the first synthetic PI3K inhibitor (PI3Kα/β/δ/γ IC_50_ = 0.55/ 16/1.6/12 μM) [32], which demonstrated chemical stability and improved selectivity against irrelevant kinase, while the selectivity towards class I PI3Ks, PI3K-related kinases was not so good.

To overcome the disadvantage of toxicity due to lack of selectivity, poor solubility, and low stability [33], derivatives with good pharmacodynamics were studied and PX-866 (4), the stable, furan-ring-opened derivatives of Wortmannin was thus identified (PI3Kα/δ/γ IC_50_ = 5.5/9.0/2.7 nM), which is currently being evaluated in phase I/II trials for the treatment of patients with advanced solid tumors.

Aller *et al* [34] discovered that (-)-epigallocatechin-3-gallate ( 5, EGCG, Ki = 0.38, 1.5, 0.81, 0.61 and 0.32 μM for PI3K α, β, δ, γ and mTOR, respectively), a major component of green tea, as well as its related catechins including CG (6, Ki=1.8/3./3.2/0.91/0.14 μM, for PI3K α/β/δ/γ and mTOR, respectively), ECG (7, Ki = 4.2/5.4/5.2/3.0/0.28 μM, for PI3K α/β/δ/γ and mTOR, respectively), GCG (8, Ki = 0.43/1.3/0.79/0.61/0.14 μM for PI3K α/β/δ/γ and mTOR, respectively) were all pan-PI3K/mTOR inhibitors. Molecular docking studies showed that EGCG was an ATP-competitive inhibitor of PI3K by binding well to the PI3K kinase domain active site.

Besides, many recent studies have demonstrated that a variety of natural products (or nutraceuticals) isolated from plants (e.g. fruits, vegetables, spices, nuts, legumes, herbs, etc.) also inhibit PI3K signaling, and exhibit potent anticancer activities. In 2013, Huang [35] briefly summarized that Apigenin, a family member of flavonoids, abundant in fruits (oranges, apples, cherries,grapes), vegetables (onions, parsley, broccoli, sweet green pepper, celery, barley, tomatoes)and beverages (tea, wine); Cryptotanshinone, one of the major tanshinones isolated from the roots of the plant Salviamiltiorrhiza Bunge (Danshen); Curcumin (diferuloylmethane), a polyphenol natural product of the plant Curcuma longa; Fisetin, a family member of flavonoids, occuring in fruits and vegetables( such as strawberries, apples, persimmons and onions); Indoles , natural compounds in cruciferous vegetables (such as broccoli, cauliflower, cabbage and brussels sprouts), especially indole-3-carbinol and it's *in vivo* dimeric product 3,3-diindolylmethane (DIM); Isoflavones, a class of flavonoid phenolic compounds, rich in soybean; Quercetin, a polyphenolic compounds, mainly from consumption of tea, onions, red grapes, and apples; Resveratrol, a natural polyphenol rich in red grapes and red wine; Tocotrienols, members of vitamin E superfamily; and many other natural products( such as caffeine , epigallocatechin gallate (EGCG, in green tea), celastrol (in traditional Chinese medicine named “Thunder of God Vine”), butein (in the stems of Rhus verniciflua, used as a food additive and as an herbal medicine in Asia), capsaicin (in chili peppers) and β-elemene (from the traditional Chinese medicinal herb Rhizoma zedoariae), etc.), have been reported to act as anticancer agents at least partly by inhibiting PI3K, Akt or mTOR activity.

## PYRIMIDINES AND QUINAZOLINES

The pyrimidine containing PI3K inhibitors have always been the most interesting area, which include most of the clinical candidates, such as GDC-0941(13), PKI-402(55), GNE-477 (66), BKM-120(90), PI-103(102), GDC-0980(107), and PF-04691502 (122), PF-06465603 (124) PF-04979064 (127). Most of the compounds in this series are PI3K/mTOR dual inhibitors, while a few exhibited PI3K isoform or kinase selectivities, which is the major goal of the development of the PI3K inhibitors currently.

In 2008, series of thieno [3, 2-d] pyrimidine derivatives were prepared and evaluated as inhibitors of PI3K p110α by Folkes *et al* [36]. The lead (9) was reported as a potent PI3Kα inhibitor, but with poor pharmacokinetic profile. Derivatives with substitution of 6-positions (10) and the replacement of phenol group (11) were synthesized. Indazoles(12) as the replacements of phenols serve as a hydrogen bond donor with Tyr836, while reduced glucuronidation and resulted in acceptable oral bioavailability. This resulted in the discovery of GDC-0941(compound 13, PI3Kα/β/δ/γ IC_50_ = 3/3.3/3/7.5 nM, mTOR IC_50_ = 0.58µM), a potent, selective, orally bioavailable inhibitor of Class I PI3K including the p110α mutant enzymes, and is currently being evaluated in human clinical trials for cancer treatment.

In 2009, series of morpholine-containing pyrazolopyrimidine analogues were synthesized and evaluated by Zask *et al* [37]. The optimization of compound 14 (PI3Kα IC_50_ = 47 nM, mTOR IC_50_ = 9.6 nM) including the phenol group bioisosteres (15), piperidine ring substituents (16) and urea analogues (17), led to the discovery of potent mTOR kinase inhibitors (mTOR IC_50_ ~ 0.08- 2450 nM) with great selectivity (~ 5-1500 fold) versus PI3Kα ( PI3Kα IC_50_ ~ 6-2000 nM).

Then they [38] investigated the effects of morpholine substitution on the potency and selectivity of pyrazolopyrimidines (18-21) by incorporating chiral, achiral methyl substituted morpholines and bridged morpholines (22-29). The result showed that these chiral morpholines led to potent mTOR inhibitors (mTOR IC_50_ ~ 0.1-100 nM) with great selectivity (~ 32-20000 fold) versus PI3Kα (PI3Kα IC_50_ ~ 35-9000 nM). Molecular modeling [38] suggested that a leucine for phenylalanine substitution in mTOR versus PI3K in the hinge regions led to a deeper pocket in mTOR relative to PI3K that could better accommodate the steric bulk of the bridged morpholine.

Meanwhile, Verheijen *et al* [39] in the same team synthesized a series of 4-morpholino- 6-aryl -1H-pyrazolo [3, 4-d] pyrimidines (30-33) (mTOR IC_50_~ 0.3-500 nM, PI3Kα IC_50_ ~ 14- 2000 nM) as mTOR inhibitors, while some 6-ureidophenyl substituents (34) led to potent dual inhibitors of mTOR and PI3Kα( selectivity ~ 0.4-3000 fold).

Liu *et al* [40] in Pfizer discovered 4-methylpteridinones (36) as orally active and selective PI3K/mTOR dual inhibitors (PI3Kα Ki ~ 2-82 nM, mTOR Ki ~ 0.85-3940 nM), with non-selective inhibitor 2-aminopyridopyrimidinone (35) as the lead. The 4-methylpteridinones were designed based on a small special pocket within PI3K and mTOR binding pocket to improve selectivity against other kinases, which was to be able to accommodate the methyl group of compound 37 and 38. Series of compounds (e.g. 39) with excellent selectivity for PI3K and mTOR were discovered. In addition, small changes in the C-6 aryl group will have profound effects on either PI3K or mTOR potency.

Nowak *et al* [41] reported the identification and optimization of pyrazolopyrimidines as mTOR kinase inhibitors (41-43, mTOR IC_50_ ~ 4-7300 nM, PI3Kα IC_50_~ 31- 6000 nM).The lead (40, mTOR IC_50_ = 215 nM, PI3Kα IC_50_ = 36 nM), a PI3K/ mTOR dual inhibitor, was identified by high throughput screening (HTS). Finally, a potent and selective mTOR inhibitor 44 (mTOR IC_50_ = 9 nM, PI3Kα IC_50_ = 1962 nM) was discovered.

Malagu et al [42] discovered a novel series of mTOR kinase inhibitors(45, 46), but the PI3K inhibitory activity was not revealed, except that of compound 47, which was 8.9µM against PI3Kα, 1000- fold less potent than that against mTOR.

In 2010, Chen *et al* [10] reported a series of 4-morpholinopyrrolopyrimidine derivatives as PI3K inhibitors, by the modification of compound 48, an imidazolopyrimidine derivative with good PI3Kα activity (PI3Kα IC_50_ = 63 nM). Followed by modification on the N5 of the imidazole ring, the 3-hydroxyl group on the phenyl ring and the N7 position (49-50), 4-ureidobenzamide derivatives with extended amino groups (51-53) were synthesized with excellent cell potency. As the most potent compound, 54 (PI3Kα IC_50_ = 0.9 nM, mTOR IC_50_ = 0.6 nM) also demonstrated *in vivo* antitumor efficacy. The replacement of the 3-hydroxy methyl group with 4-arylurea is outstanding, which not only improved metabolic stability but also increased enzyme potency and cell potency [7] .

Dehnhardt *et al* [31] described the discovery of PKI-402 (55, PI3Kα/β/δ/γ IC_50_ = 1/7/14/16 nM, mTOR IC_50_ = 1.7 nM), a novel dual PI3K/mTOR inhibitor. With imidazole- -pyrimidine 56 (PI3Kα/γ IC_50_ = 45/1134 nM, mTOR IC50 = 634 nM) as the lead, triazolopyrimidine 57 (PI3Kα/γ IC_50_ = 83/435 nM, mTOR IC_50_ = 250 nM), arylureido and 4-benzamidoureido analogues (58-59) were synthesized and evaluated. Compound 56 was identified, with excellent potency *in vitro* and *in vivo*, good physical properties and pharmacokinetic parameters.

Pecchi *et al* [43] identified 2-morpholino 6-(3-hydroxyphenyl) pyrimidine (60, PI3Kα IC_50_= 0.031 µM) as a potent and selective PI3K inhibitor initially, then analogues of 2, 4, 6-trisubstituted pyrimidine (61-63) were prepared by solid phase synthesis and evaluated (PI3Kα IC_50_ = 0.031-16 µΜ), which were approximately equipotent against α and δ isoforms, while about 10-fold less potent against the β and γ isoforms.

Heffron *et al* [44] (Genentech) discovered that, 2-aminopyrimidine derivatives replacing indazole moiety of GDC-0941 (13, PI3Kα/mTOR IC_50_ = 3/580 nM), exhibited improved mTOR inhibition and improved potency while maintaining PI3Kα inhibition. This finding was an entry into the identification of many attractive PI3K/mTOR dual inhibitors. Modification in the 6- and 7- position (64-65)of the thienopyrimidine core resulted in comparable potency (IC_50_~1-7 nM and 29-59 nM for PI3K and mTOR, respectively).The 7- methyl group was introduced to disrupt planarity and improve clearance *in vivo*. This led to the identification of GNE-477 (66, PI3Kα/β/δ/γ IC_50_ = 4/86/6/15 nM , mTOR IC_50_ =4 /21 nM), a potent dual PI3K/mTOR inhibitor with desirable pharmacokinetic properties.

Zask and Verheijen *et al* (Wyeth) previously reported [38] that bridged morpholines on pyrazolopyrimidine(67) and thienopyrimidine (68) scaffolds with apara-ureidophenyl substituent led to potent mTOR inhibitors with greater selectivity for mTOR versus PI3K than the corresponding morpholine containing analogs. They introduced the ethylene-bridged morpholine, and 4-ureidophenyl groups substituent in 4-morpholinothieno [3, 2-d] pyrimidines [45] (e.g., compound 69 and 70, moderate PI3K/mTOR dual inhibitors), which led to highly potent mTOR inhibitors (IC_50_ ~ 0.29-100 nM) with good selectivity (up to>1000-fold) over PI3Kα (IC_50_ ~ 8.3-10000 nM).

In 2010, they extended these discoveries to other scaffolds, including thienopyrimidine (71-73) and triazine (74-76) scaffold. And then, bridged morpholines (26-29) were incorporated in monocyclic triazine PI3K/mTOR inhibitors [46] , and compounds with ureidophenyl groups (76) gave highly potent and selective mTOR inhibitors (IC_50_ ~0.5-130 nM) over PI3Kα (IC_50_~24-3438 nM), as expected.

Sutherlin *et al* [47] (Genentech) discovered (thienopyrimidin-2-yl) amino pyrimidines (77-78) as pan-PI3K and pan-PI3K/mTOR dual inhibitors. Structural modification of the GDC-0941 (13, a pan-PI3K inhibitor,IC_50_ = 3/33/3/66/580 nM for PI3K α/β/δ/γ and mTOR, respectively) resulted in compound (79), a potent pan-PI3K/mTOR dual inhibitor (IC_50_ = 3.4/12/16/16/30 nM for PI3Kα/β/δ/γ and mTOR, respectively). The increased mTOR potency was presumably caused by the aminopyrimidine group, which was adjacent to Asp836 (PI3Kγ)/ Glu2190 (mTOR), and the Glu2190 was more flexible than Asp836. Then compound (80) was designed, with 4-methyl group added on the aminopyrimidine, and lacked mTOR inhibitory activity (IC_50_ = 3.5/25/5.2/15/ 750 nM for PI3K α/β/δ/γ and mTOR, respectively). The crystal structure showed that [47] this selectivity was due to the 4-methyl group, which twisted the amino pyrimidine ring out of the plane of the thienopyrimidine, and pointed toward the upper surface of the binding pocket where differences in mTOR and PI3K exist.

Venkatesan *et al* [48] reported a series of novel 2-aryl or heteroaryl substituted-4-morpholino imidazolopyrimidine derivatives (81-82) as moderate to potent dual PI3K/mTOR inhibitors (PI3Kα IC_50_ ~11-189 nM, PI3Kγ IC_50_ ~ 47-10000nM, mTOR IC_50_ ~ 51-7200 nM), which had good tumor cell growth inhibition and suppression of pathway specific biomarkers such as phosphorylation of Akt.

In 2011, Burger *et al* (Novartis) [49] discovered a series of 2-morpholino, 4-substituted, 6-heterocyclic morpholino pyrimidines (84-86, PI3Kα IC_50_ ~ 2-7740 nM) as potent PI3K inhibitors. The lead compound 83 was a potent pan class I PI3K inhibitors (PI3Kα IC_50_~50 nM) with poor pharmacokinetic properties due to the phenol group. Then the C6 phenol moiety was replaced by diverse heterocycles, and the aminopyridine turned to be the best choice, being equipotent to the phenol. After the C4 position was further optimized, pharmacokinetic and efficacy study conducted, compound 87 (PI3Kα IC_50_ < 2 nM) was identified with efficacy and suitable *in vivo* pharmacokinetic properties.

Then in their continued study [50] , C4’modified, C6 pyridyl or pyrimidyl substituted 2-morpholino 4-aminoquinolyl pyrimidines (88) were synthesized and evaluated, aiming to improve potency and reduce the *in vivo* CL values. “Incorporation of a morpholine group at the C4 position increased the aqueous solubility while maintaining potency, selectivity, and *in vivo* properties”. This led to the discovery of substituted 6-aminoheterocyclic 2, 4-bis morpholino pyrimidines (89), of which the highest soluble and the most potent compound was compound 90 (NVP-BKM120, PI3Kα IC_50_ ~ 30nM, mTOR IC_50_ ~ 4600nM) that has entered into Phase II clinical trials for the treatment of cancer.

In the design of PI3K/mTOR inhibitors from pyrido[2.3-d]pyrimidin- 7-one (91) and pteridinone (92), Liu *et al* (Pfizer) [51] introduced intra-molecular hydrogen bonding to the quinazoline motif to form a pseudo ring (intra-molecular H-bond scaffold, iMHBS), which was confirmed by the initial compound 94 (PI3Kα Ki = 18 nM, mTOR Ki = 416 nM) and 95 (PI3Kα Ki = 26 nM, mTOR Ki = 13 nM). This design resulted in potent PI3K/mTOR dual inhibitors (93, PI3Kα Ki up to 0.3nM, mTOR Ki up to 3nM).

Heffron *et al* (Genentech) [52] described two chemical series achieving PI3Kα selectivity versus PI3K*β*, which could be explained using homology model of PI3K*β*. In the thienopyrimidine series (96, PI3Kα Ki ~ 0.4-47 nM, PI3K*β* Ki ~ 7-1167 nM), the selectivity (e.g. 98 and 99) was derived from “a hydrogen bonding with Arg770 of PI3Kα that is not attained with the corresponding Lys777 of PI3Kβ”. In the benzoxepine series (97), the selectivity (e.g. 100 and 101) was due to the “electrostatic potential differences between the two isoforms in a given region”.

Using PI-103 (102) as the lead, Large *et al* [53] designed two series of trisubstituted pyrimidines, 3-hydroxyphenol analogues (103-104) and bioisosteric replacements (105) , as PI3K inhibitors. The 3-phenolic motif was replaced by three surrogate types (A, B and C), to avoid the glucuronidation *in vivo*. The most potent inhibitor was 6-aryl substitution compound 106 (PI3Kα IC_50_ =62 nM), with similar activity against PI3Kβ and δ. All three surrogate types had metabolic stabilities and inhibitory activity similar to those of parent phenols.

Sutherlin *et al* (Genentech) [54] reported the discovery of GDC-0980 (107), a potent, selective, and orally available class I PI3K/ mTOR inhibitor (PI3K α/β/δ/γ IC_50_ = 5/27/7/14 nM, mTOR Ki = 17 nM), modified from the class I PI3K inhibitor GDC-0941(13). The 2-aminopyrimidine substitution for the indazole (108) increased potency for mTOR by 20-fold. A methyl group was then added to the thienopyrimidine core (109) to lower the *in vivo* clearances and a lactic amide was used to replace sulfonamide to increase the solubility. This compound had entered clinical trials for cancer.

In 2012, Finlay *et al* (AstraZeneca) [55] described a high throughput screening approach to identify ATP competitive mTOR kinase inhibitors, starting with a modestly potent inhibitor sulfonyl morpholinopyrimidine 110 (mTOR IC_50_ = 1.41 µM, PI3Kα IC_50_ = 17.3 µM). Variation of substituents at the pyrimidine 2, 4, 6 position (111) provided compounds with higher mTOR potency (mTOR IC_50_= 0.02-100 µM, PI3Kα IC_50_ = 0.56-300 µM). The urea derivatives such as 112, exhibited the highest mTOR enzyme potency and also retained selectivity against PI3Kα (mTOR IC_50_=0.028 µM, PI3KαIC_50_ = 0.565 µM), which confirmed that the [4-(4-morpholin- 4-yl pyrimidin-2-yl) phenyl] urea motif was a privileged scaffold for mTOR and PI3K inhibition.

Koehler *et al* (Genentech) [56] designed and synthesized a novel class of potent, highly selective mTOR kinase inhibitors based upon saturated heterocycles fused to a pyrimidine core. The lead was morpholino thienopyrimidine (113), a potent mTOR inhibitor (mTOR Ki = 3 nM, 20-fold selective over PI3K). In an effort to improve the solubility and metabolic stability, by replacing the thiophene with a saturated ring, the pyrrolopyrimidines (115), 6-aza-tetrahydroquinazoline (116), 7-aza-tetrahydro quinazoline compound (117-118) were synthesized and evaluated (mTOR Ki = 1.3~1500 nM, PI3Kα Ki = 6.5 ~ >1000 nM). The result showed that the compound (114), with the phenyl ethyl urea and s-methyl morpholine hinge binder, exhibited nanomolar potency and high selectivity against PI3K and other kinases.

A novel series of 4-methylpyrido pyrimidinone (MPP) were discovered as PI3Kα/mTOR dual inhibitors by Le et al (Pfizer) [57]. The lead compound (119, PI3Kα Ki=1.56 nM, mTOR Ki =142 nM) was potent, while had poor solubility and moderate lipophilic efficiency. Then through “integration of SBDD and physical properties based optimization”, a series of analogs were designed (120, PI3Kα Ki =12.5~138 nM, mTOR Ki = 10.6~663 nM). Notably, MPP derivative (121), with a pyrazole head piece and pyrrolidinyl, exhibited good potency (PI3Kα Ki =12.5 nM, mTOR Ki =10.6 nM), desirable stability and oral bioavailability.

PF-04691502 (122), a 4-MPP derivative, exhibited potent activity against PI3K and mTOR (PI3Kα Ki = 0.57 nM, mTOR Ki = 16 nM), while “modeling studies revealed that there was still space between the terminal alcohol and the polar residues of a solvent exposed region, and no H bond interaction between MeO-pyridine and Lys 833”. Then Cheng *et al* (Pfizer) [58] designed the MPP derivatives (123) with different heteroaryl groups in the 6 position, with cis or trans- cyclohexyl in the 8 position and with a terminal alcohol, a carboxylic acid or a carboxyl amide. These compounds (123) retained potent activity (PI3Kα Ki ~ 0.31-26.4nM, mTOR Ki ~4.42-92.6 nM). PF-06465603 (124, PI3Kα Ki = 0.35 nM, mTOR Ki = 8.63nM), a metabolite of PF-04691502 with a terminal carboxylic acid, was identified.

In their search for a structurally differentiated backup candidate to PF-04691502 (122, PI3Kα Ki = 0.57 nM, mTOR Ki = 16 nM), compound (125), a tricyclic imidazo [1,5] naphthyridine, was identified as potent PI3K/mTOR inhibitor (PI3Kα/mTOR Ki = 1.41/4.51 nM), while has poor solubility, and high metabolic clearance [59] .Then tricyclic derivatives (126) were synthesized through integration of SBDD and PPBO (Physical properties-based optimization), and the most suitable compound, PF-04979064 (127, PI3Kα/γ/δ Ki = 0.130/0.111/0.122 nM, mTOR Ki = 1.42 nM ), was discovered as a backup candidate to PF-04691502.

Leahy *et al* [60] disclosed a novel series of potent and selective PI3Kγ inhibitors (131-136), based on hits 128 and 129, which were identified from HTS of ~4.6 million compounds. The sulfonyl piperazine series (131) of hit 128 had improved potency and selectivity, while with poor pharmacokinetic properties. The screening of hybrid sulfonamide derivatives (132-136) of hit 129, provided a series of promising leads with suitable pharmacokinetic properties (e.g., 130, PI3Kα/β/δ/γ IC_50_ = 435/ 2059/ 690/18 nM).

Lee *et al* [61] identified imidazolopyrimidine (137) as a modestly potent mTOR inhibitor (mTOR Ki =72 nM, PI3Kα Ki =2nM) by a HTS. To increase mTOR potency and selectivity over PI3Ks, they replaced the morpholine/aminopyrimdine of (137) with (S)-3-methyl-morpholine / ethyl phenyl urea to provide a more potent and selective mTOR inhibitor (138, mTOR Ki =72 nM, PI3Kα Ki =190nM). Using (138) as the lead, a variety of N-9-Me- imidazolopyrimidines (139, mTOR Ki = 7-12nM, PI3Kα Ki =370-390nM); N-7-Me- imidazolopyrimidines(140, mTOR Ki = 4-150nM, PI3Kα Ki >5000nM); N-5-Me- pyrrolo[3,2-d] pyrimidines(141, mTOR Ki = 6-19nM, PI3Kα Ki >3000nM), and N-1-Me-pyrazolo [4,3-d] pyrimidines (142, mTOR Ki =1-28nM, PI3Kα Ki >3000nM) were synthesize and evaluated for mTOR inhibition and selectivity against PI3K.

In 2014, Han *et al* [62] identified structurally novel and potent PI3K/mTOR dual inhibitors from a series of 2-amino-4-methylpyrido [2, 3-d] pyrimidine derivatives (145-147). As indicated by the crystal structure of PF-04691502 (122, PI3Kα /mTOR Ki = 0.57/16 nM) and GSK2126458 (143) docked into PI3Kγ, the amino-pyridopyrimidinone and quinoline formed critical hydrogen bonds with Val 882 in the hinge region. The aminopyrimidine (144) was initially synthesized and demonstrated modest inhibitory activity (PI3Kα/mTOR IC_50_ = 414/2790 nM). Then aiming to interact with Lys 833 in the affinity pocket, the methoxypyridine was modified by introducing diverse substituents in the 3-position. Compound 148 (PI3Kα/mTOR IC_50_ = 2.82 /45.8 nM) was discovered, which would be further optimized due to low permeability.

Consequently, Lin *et al* [63] from the same team reported the identification of novel 7-amino-5-methyl-1, 6-naphthyridin-2(1H)-one derivatives (149-156) as potent PI3K/mTOR dual inhibitors, by exploring the 4-methylpyridopyrimidinone (MPP), which was proven to be a potent scaffold for PI3K/mTOR dual inhibitors, such as PF-04691502 (122, PI3Kα/mTOR Ki = 0.57/16 nM). The identified representative compound (e.g. 157, PI3Kα/mTOR IC_50_= 2.42/8.55 nM) demonstrated acceptable potency, cellular activity and pharmacokinetic profile.

To discovered novel PI3Kα/mTOR inhibitors, 2-amine-4- heterocyclic aryl-disubsituted pyrido[2,3-d]- and pyrido[3,2-d] pyrimidines with (158-161)were designed by Saurat *et al* [64] . Seven promising PI3Kα/mTOR dual inhibitors (IC_50_ values <100 nM) were discovered, and two urea derivatives 162(PI3Kα/mTOR IC_50_= 58/5 nM) and 163 (PI3Kα/mTOR IC_50_ = 40/1 nM) were further developed to enhance their efficacy.

Zhu *et al* [65] reported the discovery of 7, 8-dihydro-5H-thiopyrano [4, 3-d] pyrimidine derivatives (165-166) as mTOR inhibitors, using scaffold hopping of the lead compound (164, mTOR IC_50_ =1.37 µM), a triazinehydrazone derivative. The selected compounds (mTOR IC_50_ ~ 0.8-6.93 µM)) showing equal to more potency than the lead were further evaluated for the inhibitory activity against PI3Kα (PI3Kα IC_50_ ~ 6.2-24.9 µM). SARs and docking studies showed that “the thiopyrano [4, 3-d] pyrimidine scaffold had little effect on the antitumor activities, while variations in substitutions of the aryl moieties had a significant impact on the activities and 4-OH substitution produced the best potency”.

Shao *et al* [66] designed 2-substituted-3- sulfonamino- 5- (4-morpholinoquinazolin-6-yl) benzamides (167) as bioisostere of GSK2126458 (143). Compound 168 with potent antiproliferative activity *in vitro* was selected for PI3K /mTOR enzymatic activity assay (PI3Kα/β/δ/γ IC_50_ =14/190/74/56 nM, mTOR IC_50_=65nM), Western blot assay and anticancer effects *in vivo*, and the result confirmed that these compounds could be potent PI3K inhibitors and anticancer agents. Furthermore, the docking result of 168 with PI3Kγ indicated that benzamide group could replace the complex of pyridine with water molecule in GSK2126458.

To discover dual pan-PI3K/mTOR inhibitors, Poulsen *et al* [67] generated a pharmacophore model and designed a series of novel compounds based on a purine scaffold. Three scaffolds (A-C) having a purine core substituted with a morpholine, a phenol headgroup, and a hydrophobic substituent were initially designed from three reference compounds PI-103(102), LY294002 (3) and ZSTK474 (169). Scaffold (A) was chosen for synthetic reason. The extensive SAR study of the headgroup, 8-position and 9-position substituents(170-177), utilizing the docking difference between PI3Kα and mTOR, resulted in potent inhibitors with good pharmacokinetic properties, of which, a dual mTOR/PI3K inhibitor SB2343(178, VS-5584) and a selective mTOR inhibitor SB2602 (179) progressed into clinical trial.

Dihydropyrrolopyrimidine derivative (180, PI3KαIC_50_ = 42 nM) was identified as a metabolically stable and potent PI3K inhibitor initially, while with poor oral bioavailability. To remove the H-bond acceptor and recover the water solubility, Kawada *et al* [68] designed pyridine, benzylamine and benzamide derivatives (181, PI3KαIC_50_ ~ 14-220 nM), by adding amine or amide (piperazine, morpholine) as a solubilizing group, replacing pyridine with a phenyl moiety and introducing an ortho-substituent in the phenyl group. Finally, compound 182 was identified with good pharmacokinetic profiles (oral bioavailability in monkey 8 times better than that of compound 180) and PI3Ka inhibition (PI3Kα IC_50_ = 42 nM).

In 2016, to improve the low solubility of compound (183, PI3Kα = 13 nM) by introducing a solubilizing group and ortho substituents to break molecular planarity, phenylurea derivative 184 (PI3Kα ~ 10-260 nM) were designed by Kawada *et al* [69]. Finally, compound 185 (PI3K α/β = 22/7 nM) with moderate solubility showed strong tumor growth inhibition *in vivo*.

Zhang *et al* [70] reported a series of N-(2-methoxy-5-(3-substituted quinazolin-4(3H)-one-6-yl) -pyridin-3-yl) phenyl sulfonamide (187-190) as PI3K inhibitors, using compound 186 as the lead. All compounds exhibited significant anti proliferative activities, of which, compounds 191 (PI3K α/ β/δ/γ IC_50_ = 7.3/209/106/116 nM, mTORIC_50_ = 208 nM) and 192 (PI3Kα/β/δ/γIC_50_ = 6.7/24/21/ 181 nM, mTORI_50_ = 11 4nM) displayed potent inhibitory activity against PI3K and mTOR.

## PYRIDINES, QUINOLINES, INDOLES AND INDAZOLES

Mostly, the few PI3K inhibitors based on pyridine, quinoline and indole structures reported since 2010 have been identified to be selective against PI3Kα or mTOR, including GSK2126458 (143), the most potent PI3K inhibitor with low picomolar activity.

In 2010, Barile *et al* [16] identified a novel scaffold of the 3-ethynyl-1H-indazoles (193), as multiple PI3K/PDK1/mTOR inhibitors, and discovered a PI3Kα isoform-specific compound (194), with 100-fold selectivity over the β- and γ-isoforms (IC_50_ = 0.36, 40, 10.7, 39 and 3.87 µM for PI3Kα, PI3Kβ, PI3Kδ, PI3Kγ, mTOR and PDK, respectively). The binding mode revealed that “compound 194 was deeply inserted by forming hydrogen bonds with residues in the ATP-binding site: the N-2 with Tyr836 and Asp933, the NH-1 with Asp810, NH of the pyridine ring and Val851 of the hinge region”.

Zhang *et al* [71] (Wyeth) developed a series of 5-ureidobenzofuran-3-one indoles (195-197), as potent inhibitors of PI3Kα (IC_50_~ 0.2-1 nM) and mTOR (IC_50_ ~ 0.3-1000 nM). The most potent compounds were 198 (PI3Kα/mTOR IC_50_=0.3/1 nM) and 199 (PI3Kα/mTOR IC_50_ = 0.2/0.3 nM), with a 4-[2-(dimethylamino) ethyl] methylamino amidophenyl group and 7-fluoro substituted on the indole ring. The predicted binding mode indicated that “the larger group (R1, R2) was more favorable for binding to mTOR, without affecting interactions with PI3Kα”.

Knight *et al* [72] (GlaxoSmithKline) reported the discovery of GSK2126458 (143), a highly potent, orally bioavailable PI3K/ mTOR inhibitor (Ki = 0.019/0.03/0.024/0.06/0.18/0.3 nM for PI3Kα/β/δ/γ, mTORC1 and mTORC2 respectively), as follow-up studies of GSK1059615 (200, PI3Kα Ki = 2 nM), their first PI3K clinical compound. The crystal structure of 200 indicated that larger groups (e.g. pyridine, indazole, formazaindazole, pyridylsulfonamide, arylsulfonamides), instead of the thiazolidinedione (TZD) ring, could improve potency and selectivity. Then pyridine analogues (201) were designed and GSK2126458 (143) with low picomolar activity was identified. Cocrystal structure showed that the sulfonamide group made a strong charged interaction with Lys833, which may explain the superior potency of 143.

Hong *et al* [73] identified a series of [3, 5-d]-7-azaindole analogs (202-203) as PI3Kα inhibitors, by varying groups on the 3, 5-positions of azaindole. In their pharmacophore- directed design, through fragment-based approach, 7-azaindole possessing both H-donor and H-acceptor was selected as a scaffold, and the pyridyl sulfonamide pharmacophore was introduced at C5-position to interact with the back pocket (DFG-motif, gate keeper and catalytic lysine). These 7-azaindole derivatives exhibited modest to good activity in cellular proliferation (PI3KαIC50=3~5200nM) and in apoptosis assays.

In 2011, Kim *et al* [74] designed and synthesized a new series of imidazo [1, 2-a] pyridine derivatives (204-205) as PI3Kα inhibitors (PI3Kα IC_50_ ~ 0.2-720 nM). With the selected imidazopyridine as the hinge linker binder and pyridyl sulfonamide as the back pocket group, they explored the structures by attaching diverse groups at the C3 position to fill the ribose pocket. The SAR results showed that some moieties (e.g., ester, nitrile, oxadiazole, tetrazole, and pyridine) at the C3 position profoundly influenced PI3Kα binding affinity (e.g. compound 206-208, PI3Kα IC_50_~ 1 nM).

In 2011, Liu *et al* [75] discovered benzonaphthyridinone analogs (210) as potent and selective small molecule mTOR inhibitors, by replacing the metabolically labile 4-amino-phenylpiperazine moiety of mTOR inhibitor Torin1 (209, PI3Kγ/mTOR EC_50_= 1800/2 nM) with a phenyl ring. Further modification resulted in a new mTOR inhibitor (231, PI3Kγ/mTOR EC_50_ = 1000/5 nM) that had significantly improved stability, as well as retained potency and selectivity.

As their continued study, they [76] discovered Torin 2 (213, PI3K/mTOR EC_50_ = 200/0.25 nM) as a potent, selective, and orally available mTOR inhibitor, utilizing a focused medicinal chemistry approach guided by cellular assays and pharmacokinetic and pharmacodynamic assays of compounds 212. The co-crystal structure revealed that the aminopyridine group formed three hydrogen bonds in the hydrophobic pocket.

Nishimura *et al* [77] reported the discovery of a series of substituted quinolines and quinoxalines derivatives (215-217), as potent PI3K/mTOR dual inhibitors (e.g. 203, PI3Kα Ki = 0.6 nM) with excellent pharmacokinetic properties and *in vivo* efficacies, using compound (214) as the lead. Initially, analogues with 6, 6-bicyclic heterocycles (quinoline, isoquinoline, quinoxaline, quinazoline, cinnoline, and naphthyridine) were designed, to replace the benzothiazole, as hinge linker binder. Then by incorporating suitable substituents at the 4-position of the quinoline or the 3-position of the quinoxaline rings, excellent cellular potencies were achieved, which indicated that the ribose pocket of the enzyme can be effectively utilized in optimizing both the potency and the physicochemical properties of PI3K inhibitors [77] .

In 2013, Li *et al* [78] synthesized HS-106 (219, PI3Kα IC_50_=11nM), by screening the above chemical library of imidazopyridine derivatives [74]. They found this compound “suppressed breast cancer cell proliferation and induced apoptosis by inducing apoptosis and suppressing angiogenesis”, which could be a potential drug for breast cancer treatment.

In order to design and optimize 3-pyridine heterocyclic derivatives as PI3K/mTOR dual inhibitors, molecular docking and 3D-quantitative structure–activity relationship (3D-QSAR) studies based on the ligand alignment and receptor alignment were applied using the comparative molecular field analysis (CoMFA) and comparative molecular similarity indices analysis (CoMSIA) were carried out by Yang *et al* [79] . Highly accurate and predictive 3D-QSAR models for designing new PI3K/mTOR inhibitors were constructed (Skelton 220-222), which would be useful for predicting activity and guiding the ligand modification of PI3K/mTOR inhibitors.

Tsou *et al* (Weyth) [80] discovered 2-(4-substituted-pyrrolo [2, 3-b] pyridin-3-yl) methylene- 4-hydroxybenzofuran-3(2H)-ones (224-231) as potent and selective mTOR inhibitors. With the indole bearing 4, 6-dihydroxy benzofuranone (223) as the lead, a variety of 4-substituents, including 4-hydroxy phenyl, 4-benzamides and 4-piperidine amides were introduced on the indole. Also, since phenolic OH group was metabolically liable, one of the two hydroxyl groups was selectively removed. These optimizations generated subnanomolar, selective mTOR kinase inhibitors (mTOR IC_50_ = 0.39-180 nM, PI3Kα IC_50_ = 16-5895 nM) with low nanomolar cellular activity.

In 2015, Lv *et al* [81] synthesized novel 4-alkynyl-quinoline derivatives (232-234) as PI3K/mTOR dual inhibitors (PI3Kα IC_50_~1.63-300nM) by modification of GSK2126458 (143). “To improve the water solubility and explore potential interactions with residues in the ribose pocket (e.g. Lys802 and Ala805)”, the pyridazine of GSK2126458 was replaced with a hydrophilic substituent, while an alkyne was employed as a linkage between this hydrophilic group and the quinolone core. The target compounds showed potent PI3Kα inhibitory activities (PI3Kα IC_50_~1.63-300nM) and good anti-proliferative effects. Compound 235, the 4-hydroxylpiperidine derivative, was further identified as a potent PI3K/mTOR dual inhibitor (PI3Kα/β/δ/γ IC_50_ = 1.63/6.91/2.14/0.38 nM, mTORIC_50_ = 3.26 nM).

### Pyrazines and quinoxalines

Given that the pan PI3K inhibitors XL-765 (236) and XL-147 (237) in clinical trials was quinoxaline derivatives, few PI3K inhibitors based on pyrazine and quinoxaline core have been developed, while with limited potency mostly.

In 2011, Wu *et al* [82] synthesized a series of novel 2-arylamino-3-(arylsulfonyl)quinoxalines (238) through a newly developed approach from XL-765 (236, PI3K α/β/δ/γ IC_50_=39/113/43 /9 nM ) and XL-147 (237, PI3K α/β/δ/γ IC_50_ = 39/383/36 /23 nM).The most potent compound (239, PI3Kα IC_50_ = 0.07 µM) validated the potential of 2-arylamino- 3- (arylsulfonyl) quinoxaline series for cancer treatment by targeting PI3Kα.

Wu *et al* [83] identified novel piperidinylquinoxalines (242) and piperazinylquinoxalines (243-246) as PI3Kα inhibitors, with previously identified morpholinoquinoxaline derivative 240 (PI3Kα IC_50_=0.44 µM) and piperidinylquinoxaline derivative 241 (PI3Kα IC_50_= 0.025 µM) as the lead. Modification at the 2-position of the quinoxaline scaffold and 4-substituent of phenylsulfonyl moiety led to novel PI3Kα inhibitors with good to potent inhibitory activity (up to 24 nM).

In 2011, Mortensen *et al* [84] reported the discovery of the imidazo [4, 5-b] pyrazin-2-one series (247) as selective mTOR kinase inhibitors (e.g., 248 and 249). As the continued studies, through ring-expansion of the imidazo-ring by insertion of a methylene unit, they [85] discovered a new series of potent and selective mTOR kinase inhibitors (250) with exquisite kinase selectivity (mTOR IC_50_ ~ 2-176 nM, PI3Kα IC_50_> = 526 nM), which led to the identification of CC214-2 (251, mTOR IC_50_=0.002 nM, PI3Kα IC_50_ =1.38 nM), an orally available mTOR kinase inhibitor with demonstrated anti-tumor activity in mice.

In 2012, Martinez *et al* [86] described a novel series of 8-morpholinyl-imidazo [1,2-a]pyrazines (252-254) bearing an N atom in bridge head position as PI3K inhibitors, which showed good potency against PI3Kδ and α, with improved selectivity against mTOR kinase. The inhibitory activity of the most potent compound (255) was 2.8nM, 60nM and >10 µM, against PI3Kδ, α and mTOR, respectively. Then a variety of PI3K inhibitors (256-258) were explored, by replacing the 4-indazol moiety with heteroaryls, C2 position with additional amino alkyl substituents and C3 position with simple substituents such as bromine and methyl [87] . Finally, ETP-46321(259) has identified as a potent and orally available PI3K α/δ inhibitor (PI3K α/β/δ/γ IC_50_ = 2.3/170/14.3/ 17 nM) with high selectivity over mTOR ( mTOR IC_50_= 2.4 µΜ) and 288 representative kinases.

### Azoles

Azoles (including diazole, triazole, thiazole, oxadiazole, etc.), five-member heteroaryls of pyrimidine isosteres, are also the proper scaffolds for PI3K inhibitors. Compounds in this series always achieved isoform selectivity, e.g. PI3Kβ selective inhibitor SAR260301 (332, PI3K α/β/δ/γ IC_50_ = 1539/23/469/10000 nM), PI3Kα selective inhibitor BYL719 (341, PI3Kα/β/δ/γ IC_50_ = 5/1200/290/250 nM) and GDC-0326 (310, PI3Kα/β/δ/γ IC_50_=0.2/26.6/4/10.2 nM), as well as PI3Kα/δ/γ inhibitor GDC-0032 (309, PI3Kα/β/δ/γ IC_50_=0.29/9.1/0.12/0.97 nM).

In 2008, Alexander [88] identified 4-(1, 3-thiazol-2-yl) morpholine derivatives (261) as potent and selective PI3K inhibitors by the modification of compound 260 (PI3K α/β/δ/γ IC_50_ = 1333/ 693/701/3453nM). The most potent compounds lactam 263(PI3K α/β/δ/γ IC_50_ = 59/1006/18/31 nM) showed similar potency to the ketone 262 (PI3K α/β/δ/γ IC_50_ = 51/ 1157/35/49nM), while 263 exhibited better *in vivo* pharmacokinetic profiling based on its superior solubility.

In 2011, Bengtsson *et al* [89] patented the 5-heteroaryl thiazole derivatives (264) or a pharmaceutically acceptable salt and their use as PI3K and/or mTOR inhibitors. In general, compounds of the invention possessed PI3K inhibitory activity with PI3Kγ IC_50_ ~ 0.1-40µM and PI3Kα~0.1-4.5µM.

Angelo *et al* [90] identified benzothiazole compound 265 (PI3Kα Ki = 53 nM, mTOR Ki > 25 μM) as an initial hit from HTS, and the crystal structure suggested that the ribose pocket might accommodate larger groups than pyrimidine. Extensive SAR studies, including the link atom (sulfur, oxygen and nitrogen) and the pyridine replacing pyrimidine (266-268), led to the sulfonamide 269 (PI3Kα Ki = 38 nM, mTOR Ki = 269 nM) for as an early lead, with high *in vitro* and *in vivo* clearance. Subsequent modifications, including the central ring and the substitution (270-271), led to chloropyridine 272 (PI3Kα Ki<1 nM, mTOR Ki = 2.1 nM). Further phenyl sulfonamide SAR studies (273) optimizing *in vitro* clearance led to the identification of as a potent PI3K/mTOR dual inhibitor 274 (PI3Kα Ki =1.2 nM, mTOR Ki = 2.0 nM), with low clearance and high oral bioavailability.

Liu *et al* (Pfizer) [91] discovered tetra-substituted thiophenes as highly selective PI3K inhibitors, with compound 275 (PI3Kα Ki = 230 nM) as the lead, which was initially optimized by replacing the free carboxylic acid moiety with carboxylic amide (276, PI3Kα Ki =13 nM). As nitrogen atom could form H-bond binding with a water molecule in the ATP binding site, different amide bioisosteres of compound 276 were designed (277-281), of which compound 277 with a 1, 2, 4-triazole group stand out (PI3Kα Ki = 1.7 nM, mTOR Ki = 434 nM). C-4 phenyl moiety was then replaced by diverse aryl and heteroaryl groups to maximize the mTOR selectivity. Finally, very potent compounds 282 (PI3Kα Ki=0.35 nM, mTOR Ki=2470 nM) and 283 (PI3Kα Ki=0.6 nM, mTOR Ki = 1440 nM) with excellent selectivity over mTOR (up to 7000-fold) was discovered, which demonstrated good potency *in vitro* and *in vivo*, as well as the desired pharmacokinetic properties.

“Compound (284), a potent PI3Kα/mTOR inhibitor (PI3Kα IC_50_ = 1.2 nM, mTOR IC_50_ = 2.0 nM) *in vitro* and *in vivo*, was found to undergo deacetylation on the 2-amino substituent to yield compound 285 (PI3Kα Ki = 5.6 nM, mTOR IC_50_ = 84 nM)”. To reduce or eliminate this metabolic deacetylation, Stec *et al* [92] examined a variety of 6, 5-hetero-cyclic analogues (286) as an alternative to the benzothiazole ring. Finally, imidazopyridazine (287, PI3Kα IC_50_=1.4 nM, mTOR IC_50_ = 0.4 nM) was discovered, which exhibited similar *in vitro* potency and *in vivo* efficacy relative to the lead (284), while was more metabolically stable.

In 2012, Bruce (Novartis) *et al* [93] elaborated the progression from a pan-PI3K lead molecule (288, PI3K α/δ/γ /β Ki= 0.11, 0.11, 0.21, 1.43 µM) to α, δ and γ isoform selective Class I PI3K inhibitors (289-293), which was urea derivatives using parallel synthesis to combine amines (RR’NH) with selected aminothiazole scaffolds. These inhibitors with good isoform selectivity and cellular activity “would be pharmacological tools for elucidating the relative contributions of individual isoforms in PI3K signaling pathways”.

Lin *et al* (GlaxoSmithKline) have reported [94, 95] the discovery of imidazopyrimidinone (294, PI3Kα/β/γ/δ IC_50_= 2.0/0.001/0.008/1 µM) and triazolopyrimidinone (295, PI3Kα/β/γ/δ IC_50_= 0.32/0.0003/0.004/0.06 µM), as novel potent PI3Kβ selective inhibitors, while with poor pharmacokinetic profile. Then they designed [96] thiazolopyrimidinones (296), with a substituted benzyl group at the N1-position to induce the selectivity-pocket formed by Met-779 and Trp-787, a morpholine as the hinge binder and a carbonyl group to interact with the back-pocket. These compounds demonstrated potency (PI3Kβ IC_50_ = 0.05-790 µM) and good selectivity ( PI3Kβ selectivity >10 fold), and compound 297 (PI3Kα/β/γ/δ IC_50_= 2.5/0.0006/0.020/0.79 µM) emerged as a potent, selective and orally bioavailable PI3Kβ inhibitor.

Compound 298 was identified as an initial hit (PI3Kγ IC_50_ = 5 nM) through HTS by Oka *et al* [97] and the docking mode indicated that nitrogen atoms in the acetylaminothiazole formed hydrogen bonds to the hinge Val882 ,while the benzoic acid moiety interacted with Lys807 and Lys833. Thus, they optimized the central heterocycles as the replacement of thiazole and identified oxazole derivative 299 (PI3Kγ IC_50_ = 12 nM) as the lead for further optimization. A novel series of 2-aminothiazole-oxazole derivatives (300) were synthesized and evaluated as PI3Kγ inhibitors (PI3Kγ IC_50_~3-346 nM), of which the trifluoroethyl and tert-butyl derivatives displayed good enzymatic and cellular activities.

Peterson *et al* [98] identified new imidazopyridine and imidazopyridazine scaffolds (303) that demonstrated superior mTOR inhibition and selectivity, starting from the previously reported triazine-benzimidazole (301, mTOR IC_50_= 23 nM, PI3Kα IC_50_ = 798 nM) and triazine- imidazopyridine(302, mTOR IC_50_= 12 nM, PI3Kα IC_50_ = 590 nM). To capping sites of glucuronidation(the pyrazole NH) and indroducing a ribose substituent to improve pharmacokinetics and potency, the imidazopyridazine core and substitution of the linker-binder pyrimidine were explored, which resulted in potent and selective mTOR inhibitor (303, mTOR IC_50_ ~2-147nM, PI3Kα IC_50_ ~47-12500nM), with improved *in vivo* clearance.

In 2013, Morales *et al* [99] explored the 5-morpholino-7H thieno[3,2-b] pyran-7- ones (304) as potential PI3K inhibitors, with thiophene as the bioisostere of the phenyl ring of the classic pan-PI3K inhibitor LY294002. This series have improved potency with PI3Kα selectivity (e.g., 305, PI3Kα/β/γ/δ IC50= 34/214/960/158 nM), while in some cases, displayed an unexpected PI3Kδ selectivity (e.g. 306, PI3Kα/β/γ/δ IC50= 714/1750/27/1170 nM). The cell-based assay of these compounds also demonstrated potent cell growth inhibitory activity.

Then Ndubaku *et al* [100] (Genentech) derived a set of imidazobenzoxazepin compounds, by modification of compound 307, which displayed good *in vitro* potency (PI3Kα IC_50_<0.5 µΜ), while had low solubility and high *in vivo* unbound clearance. These imidazobenzoxazepin derivatives (308, PI3Kα IC_50_~ 0.1- 21.1 nM) had better tumor growth inhibition *in vivo*, and GDC-0032 (309, PI3Kα/β/δ/γ IC_50_=0.29/9.1/ 0.12/ 0.97 nM) was identified, which was undergoing clinical development for use in PI3K-related cancers. Then in 2016, they reported [101] the discovery of a series of PI3Kα-specific inhibitors, which obtained PI3Kα-selectivity through interactions with a non-conserved residue. Optimization of properties led to the identification of the clinical candidate GDC-0326 (310, PI3Kα/β/δ/γ IC_50_=0.2/26.6/4/10.2 nM).

Staben *et al* [102] (Genentech) had disclosed HTS derived thienobenzoxepin series (311) with aniline amide substituents as PI3K inhibitors, while the aniline amide was undesired and contributed to high clearance. To improve the clearance, stability and potency through interactions with the affinity pocket, they replaced he aniline amide with heterocyclic amide isosteres (312-320). Overall, simple branched alkyl substituted triazoles had better properties than those halo aryl substituted derivatives. The replacement of ‘cis’N-methyl aniline amides led to compound 321 (PI3Kα/β/γ/δ IC_50_ = 4.0/29/2.2/3.9 nM), a potent and selective PI3K inhibitor with high permeability, solubility and bioavailability.

To improve selectivity over PI3Kβ and decrease the high clearance due to the amide hydrolysis of compound 322(PI3Kα/β/γ/δ IC_50_ =4.0/29/2.2/3.9 nM), they [103] further optimized the 2-triazolyl-benzoxepin 8-substitution, and replaced thiophene with other five-membered-heteroaryls (323-328, PI3Kα IC_50_ ~ 0.04- 1.9 nM, PI3Kβ selectivity~ 1.1-3160 fold). Finally, PI3Kβ-sparing inhibitor, compound 329 (PI3Kβ Ki/PI3Kα Ki ~ 57 fold, PI3Kα/β/γ/δ Ki ~ 0.27 /15 /0.55/ 0.61 nM) was discovered with a suitable pharmacokinetic profile. Furthermore, the binding mode revealed that “the selectivity might be due to difference in the conformation of a tryptophan residue present in all isoforms (α TRP780, β TRP781, δ TRP760, γ TRP812)”,which presented a new structure-based hypothesis for reducing inhibition of the PI3Kβ isoform while maintaining activity against α, δ and γ isoforms.

The indoline amide (331), replacing the aniline of compound (330) with a secondary amine, displayed potent and selective PI3Kβ inhibition (IC_50_ = 460 nM, 4 nM, 28 nM and>10 μM for PI3Kα, β, δ and γ, respectively), but had poor aqueous solubility due to a strong network of hydrogen bond and hydrophobic interactions. To improve the solubility, 2-methyl was introduced on the indoline to yield different substituted pyrimidone indoline amides (332, IC_50_~85-10000nM, 1-407nM, 2-4135nM, 1131-10000nM for PI3Kα, β, δ and γ, respectively) by Certal *et al* [104] in 2014. Finally, compound 333 (SAR260301) was discovered as a potent and soluble PI3Kβ inhibitor (PI3Kα/β/δ/γ IC_50_ = 1539/23nM/469/10000 nM), which entered clinical trial in patients with advanced cancer.

Meanwhile, a novel thiazole carboxamide series was designed (e.g., compound 335, IC_50_ = 8280, 45 nM, 227 nM and >10,000 nM on PI3Kα, β, δ and γ, respectively) by a fragment based rescaffolding [104] , starting from pyrimidone 334 (PI3Kβ selective inhibitor, IC_50_ = 10000 nM, 42 nM, 118 nM and >10000 nM for PI3Kα, β, δ and γ, respectively). Then a series of morpholino thiazole derivatives were synthesized as PI3Kβ selective inhibitors (336, IC_50_ ~395-10000 nM, 8-340nM, 58-2723 nM and >4203 nM for PI3Kα, β, δ and γ, respectively) with suitable properties.

Collier *et al* [105] discovered a series of C6 substituted benzothiazole and urea analogues (338-339, PI3Kγ Ki~1-770nM, PI3Kα Ki~7-4000nM) as PI3Kγ selective inhibitors, evoluted from a reported phenylthiazole pan-PI3K inhibitor PKI-93 (337). From the X-ray crystallography of compound 340 (PI3Kγ Ki = 7 nM, PI3Kα Ki = 105 nM) bound to PI3Kγ, they found that the propylimidazole group occupied a previously unreported hydrophobic cleft adjacent to the ATP binding site and residue differences (Gly829 and Ala885 in PI3Kγ) in this region caused the PI3K isoforms selectivity.

Gerspacher *et al* [106] (Novartis) converted the 5-(pyridin-4-yl)thiazol-2-amino bicyclic core scaffold of BYL719 (341) into novel 4H-thiazolo[5’,4’:4,5 ]pyrano [2,3-c]pyridine tricyclic scaffold ( 342), via an oxygen as the linker. Activity result showed that 343 (PI3Kα/β/δ/γ IC_50_ = 5/670/220/200 nM) exhibited similar or better biochemical potency, selectivity against PI3Ka and cellular activity, as well as favorable pharmacokinetic properties due to enhanced solubility, compared to noncyclized analogs BYL719 (341, PI3Kα/β/δ/γ IC_50_ = 5/1200/290/250 nM).

### Triazines

ZSTK474 (169), the first triazine derivatives as a potent pan class I PI3K inhibitor, was reported in 2006. With the liability of synthesis and suitable position of the substituent, PI3K inhibitors based on the triazine or triazine-benzimidazole core structure has been continued [107], such as PKI-587(348), PKI-179 (350).

In 2010, Richard *et al* [108] (Wyeth research) identified a variety of potent triazine mTOR inhibitors containing the (R)-3-methyl morpholine moiety and a pyridylureidophenyl group, which demonstrated good selectivity (greater than 500-fold) over the related PI3Kα (344-346, mTOR IC_50_ ~ 0.2-3.6 nM, PI3Kα IC_50_ ~ 41-1894nM). SAR studies revealed that “the addition of basic amines at the 4-position of the ureidophenyl ring was well-tolerated and offered the opportunity to develop inhibitors with improved physicochemical properties, while amide derivatives at the 4-position of the arylureidophenyl ring resulted in reduced selectivity over PI3Kα but enhanced cellular activity”.

Venkatesan *et al* [48] (Pfizer) discovered that a series of bis(morpholino-1,3,5-triazine) derivatives bearing bis-aryl ureas (347) are potent dual PI3K/mTOR inhibitors. They also found that the amide bearing water solubilizing groups (eg, N (Me)2-piperidine,methylpiperazine, pyrrolidino-piperidine) enhanced potency, due to the increased H-bond accepting ability. Finally, compound 348 (PKI-587, IC_50_ = 0.4, 5.4, 1.6 nM for PI3Kα, PI3Kγ and mTOR, respectively) was identified as a highly efficacious PI3K/mTOR inhibitor *in vitro* and *in vivo*, which entered clinical trials as a single agent for i.v. administration.

However, PKI-587(348) could not be administrated orally because of poor permeability, low clog P and high molecular weight. Hence, to obtain an orally efficacious PI3K/mTOR inhibitors by increasing the clog P and to lowering the molecular weight, a series of mono-morpholino 1, 3, 5-triazine derivatives bearing a 3-oxa-8-azabicyclo [3.2.1] octane were designed [109] (349, PI3Kα IC_50_ ~7-85nM, PI3Kγ IC_50_ 44-717nM, mTOR IC_50_ ~0.32-93.5nM). An orally efficacious dual PI3K/mTOR inhibitor, compound 350 (PKI-179, IC_50_ = 8, 74, 0.42nM for PI3Kα, PI3Kγ and mTOR, respectively) with lower molecular weight was discovered. Moreover, the active metabolite of PKI-587 was determined to be 351 (IC_50_ = 4, 33, 0.8nM for PI3Kα, PI3Kγ and mTOR, respectively).

In 2011, based on the triazolopyrimidine (55, PKI-402) and triazine (348, PKI-587) scaffold as highly efficacious dual PI3K/mTOR inhibitors, Dehnhardt et al [110] (Pfizer) designed the novel 2-oxatriazines series (352-353), which also exhibited excellent potency and good metabolic stability. The most potent compound 354(PI3Kα IC_50_ = 0.2 nM, mTOR IC_50_ = 0.7 nM) showed an *in vitro* profile comparable to PKI-587.

Peterson *et al* [111] discovered triazine-benzimidazoles as selective mTOR inhibitors, using compound 355 (PI3Kα IC_50_=0.32 µM, mTOR IC_50_ =0.097 µM)) as the lead. The synthesized triazines provided broader kinase selectivity and improved potency (356-358), with diverse phenol bioisosteres to modify the affinity pocket binding moiety and the triazine linker-binder replacement to improve the pharmacokinetic properties. Compound 359 (PI3Kα IC_50_ = 2.2 µM, mTOR IC_50_ = 0.081 µM) exhibited superior selectivity to other compounds, “with 200-fold selectivity over PI3Kα, and greater than 100-fold selectivity over the other PI3K isoforms”.

As ZSTK474 (169, PI3Kα/β/δ/γ IC_50_ = 8.6/44/5/49 nM) had poor aqueous solubility which limited the development of an amorphous formulation [112], Rewcastle *et al* [113] explored the 2-substituted, 4-, 5- and 6-substituted and pyrimidine analogues of ZSTK474, to discover more soluble inhibitors (360-361). They found that substitution at the 4 and 6 positions of the benzimidazole ring generated highly potent PI3K inhibitors (e.g., compound 362, PI3Kα IC_50_ = 0.22 nM) with good pharmacokinetics and efficacy *in vivo*, while still had poor solubility properties.

In 2012, Smith *et al* [30] synthesized a series of highly selective class I PI3K inhibitors, starting from the potent PI3K/mTOR dual inhibitor 363 (PI3Kα Ki = 350 nM, mTOR IC_50_ = 93 nM) with poor pharmacokinetic properties due to the glucuronidation of the phenolic substituents and extensive metabolism of the benzimidazole *in vivo*. As “pyridylpyrimidine and pyridylpurine scaffolds had been demonstrated to have good *in vivo* properties”, scaffold 364 was explored, with additional substituents to interact with the ribose pocket (Ki values <10 nM against PI3Kα, PI3Kβ, PI3Kγ, PI3Kδ and>1000-fold selectivity against mTOR). Finally, compound 365 (PI3Kα/β/δ/γ Ki = 9/5/2/4 nM, mTOR IC_50_ = 4800 nM) was discovered, with excellent selectivity over mTOR and related kinases.

In previous studies, Wurz *et al* [90] disclosed sulfonamide benzothiazole derivatives (e.g. 366, PI3Kα Ki = 1.2 nM, mTOR IC_50_ = 2.1 nM) as potent PI3K/mTOR dual inhibitor, while had low solubility. Meanwhile, another series of PI3K inhibitors (e.g. 367, PI3Kα Ki = 9.1 nM, mTOR IC_50_ = 4.7 µM) with moderate *in vivo* pharmacokinetic properties, was identified. Then structure 368 was designed by a hybrid strategy [113], which underwent modification to the sulfonamide of the affinity pocket binding motif (R1) and the ribose pocket binding motif (R2). A novel series of 4-amino-6-methyl- 1, 3, 5- triazine sulfonamides were discovered as PI3K inhibitors (PI3Kα Ki~3.5-161 nM).

In 2013, Pinson *et al* [115] identified a series of amino acyl-triazine derivatives (369) as potent and isoform selective PI3Kβ inhibitors, by modifying the pan-PI3K inhibitor ZSTK474. The selectivity based on the stereochemistry, with L-amino acyl derivatives preferring PI3Kβ, while their D-congeners favored PI3Kδ (PI3Kα IC_50_~42-10000 nM, PI3Kβ IC_50_ ~31-10000 nM, PI3Kδ IC_50_ ~26-3600 nM, β/δ selectivity~0.007-34). This could be explained by interaction with the non-conserved binding site residue ASP862 of PI3Kβ, which provided an alternate mechanistic basis for selectivity.

### Others

To discover novel PI3Kγ inhibitors as anticancer agent, Taha *et al* [116] explored the pharmacophoric space of PI3Kγ via diverse inhibitors and used CATALYST-HYPOGEN to identify high quality binding model in 2014. Then QSAR model was assessed within training inhibitors (78 collected PI3Kγ inhibitors, scaffold 370-378) and two associated models were validated by screening for new PI3Kγ ligands. 19 NCI hits (379-397) exhibited good to moderate potencies against PI3Kγ (IC_50_ =105-9157nM) *in vitro*, which suggested that “the combination of pharmacophoric exploration and QSAR analysis could be useful to find new and diverse PI3Kγ inhibitors”.

## CONCLUSION

Unregulated activation of the PI3K/Akt/mTOR pathway is a prominent feature of many human cancers and PI3K is activated or over-expressed in all major cancers. This makes PI3K as one of the most attractive anticancer targets, which may even circumvent drug resistance to current chemotherapies proved by preclinical and clinical evidences. The discovery of PI3K inhibitors brought a lot of promising compounds as drug candidates, a dozen of which have been advanced into preclinical or clinical trials for cancer treatment. Furthermore, the first approved PI3K inhibitor, Idelalisib (p110δ selective) has already been used for the treatment of various hematological malignancies. However, there are many issues remained to be addressed.

Currently, the key point for the further development of PI3K inhibitors is selectivity. Much effort has been made to the development of class I PI3K inhibitors that exhibit sufficient isoform- selectivity and cross-kinase selectivity, with the help of the elucidation of the X-ray crystal structures of PI3K isoforms and those of their complexes with diverse inhibitors. As each PI3K isoform has its own function and is correspondingly involved in various diseases, it was assumed that the isoform-specific PI3K inhibitors may obtain lower toxicity, better tolerability and safety, while the pan- PI3K inhibitors could offer enhanced therapeutic efficacy. Likewisely, the PI3K/mTOR dual inhibitors was considered to be more effective by delivering a powerful two-spot inhibition of the pathway and have the advantage of being less susceptible to PI3K drug resistance. “Will the isoform-specific inhibitors be more tolerable than pan-PI3K inhibitors”, “Whether the dual inhibition of PI3K and mTOR is superior to inhibiting PI3K alone”, “How to find the proper balance between the safety (only through kinase selectivity) and the therapeutic efficacy” are still questions remained to be addressed. And the answer will not be known until the completion of ongoing clinical trials.

## Abbreviations

PI3K: phosphatidylinostitol-3-kinase
Akt: protein kinase B, PKB
mTOR: mammalian target of rapamycin
PTEN: phosphatase and tensin homologue deleted on chromosome ten
RTK: receptor tyrosine kinases
PIP2: phosphatidylinositol 4, 5-bisphosphate
PIP3: phosphatidylinositol 3, 4, 5-triphosphate
PDK1: phosphatidyl inositol 3-dependent kinase 1
mTORC1: mTOR complex 1
mTORC2: mTOR complex 2
4E-BP1: 4E-binding protein 1
MDR1: multidrug resistance protein-1
PIKKs: PI3K-related kinases
DNA-PK: DNA- dependent protein kinase
GPCR: G-protein-coupled receptors
SAR: Structure Activity Relationship
SBDD: Structure-based drug design
QSAR: Quantitative Structure-Activity Relationship
HTS: high throughput screening
